# Characterization and improved properties of Glutamine synthetase from *Providencia vermicola* by site-directed mutagenesis

**DOI:** 10.1038/s41598-018-34022-5

**Published:** 2018-10-23

**Authors:** Wu Zuo, Leitong Nie, Ram Baskaran, Ashok Kumar, Ziduo Liu

**Affiliations:** 1 College of Food and Bio-science and Technology, Wuhan Institute of Design and Science, Wuhan, 430205 P.R. China; 2 College of Life Science and Technology, State Key Laboratory of Agricultural Microbiology, Huazhong Agricultural University, Wuhan, 430070 P.R. China; 3 Department of Biotechnology and Bioinformatics, Jaypee University of Information Technology, Waknaghat, Solan, 173234 Himachal Pradesh India

## Abstract

In this study, a novel gene for Glutamine synthetase was cloned and characterized for its activities and stabilities from a marine bacterium *Providencia vermicola* (*Pve*GS). A mutant S54A was generated by site directed mutagenesis, which showed significant increase in the activity and stabilities at a wide range of temperatures. The *K*_m_ values of *Pve*GS against hydroxylamine, ADP-Na_2_ and L-Glutamine were 15.7 ± 1.1, (25.2 ± 1.5) × 10^−5^ and 32.6 ± 1.7 mM, and the *k*_cat_ were 17.0 ± 0.6, 9.14 ± 0.12 and 30.5 ± 1.0 s^−1^ respectively. *In-silico-*analysis revealed that the replacement of Ser at 54th position with Ala increased the catalytic activity of *Pve*GS. Therefore, catalytic efficiency of mutant S54A had increased by 3.1, 0.89 and 2.9-folds towards hydroxylamine, ADP-Na_2_ and L-Glutamine respectively as compared to wild type. The structure prediction data indicated that the negatively charged pocket becomes enlarged and hydrogen bonding in Ser54 steadily promotes the product release. Interestingly, the residual activity of S54A mutant was increased by 10.7, 3.8 and 3.8 folds at 0, 10 and 50 °C as compared to WT. Structural analysis showed that S54A located on the loop near to the active site improved its flexibility due to the breaking of hydrogen bonds between product and enzyme. This also facilitated the enzyme to increase its cold adaptability as indicated by higher residual activity shown at 0 °C. Thus, replacement of Ala to Ser54 played a pivotal role to enhance the activities and stabilities at a wide range of temperatures.

## Introduction

*Providencia vermicola*, is a marine bacterium isolated from juveniles of the entomic pathogenic nematode *Steinernema thermophilumin*^1^. Glutamine synthease (GS, L-glutamate: ammonia ligase, ADP-forming, EC 6.3.1.2)^2,3^ is an important enzyme in nitrogen metabolism which catalyses the synthesis of glutamine using ammonia produced by nitrate reduction, amino acid degradation and photorespiration^4^. It plays an important role in the metabolic pathways of marine bacteria found in oligotrophic oceans. Several researchers have studied the biological role, physico-chemical properties, and kinetic properties of GS from different sources^5–9^. The phosphinothricin (PPT) and methionine sulfoximine (MetSox) are the inhibitors of GS activity, which tightly bound to its active site^10–12^. Thus, GSI may play important role as bio-pesticide with potential usage in agricultural industry. Recently, the production of theanine using glutamine synthetase become more attractive and researchers are attempting to increase the total catalytic efficiency of GS by directed evolution, site directed mutagenesis for the synthesis of theanine in the industrial scale^13–20^. In order to improve the catalytic activity and stability of industrially important enzymes directed evolution techniques^21–24^ and various immobilization strategies were used^25–27^. The directed evolution is widely employed to generate mutants with increased catalytic activity and to investigate the role of particular amino acid residues in catalytic behaviour^28–30^. The commonly used techniques in directed evolution to improve the enzyme activity were error-prone PCR, DNA shuffling and site-directed mutagenesis^12,21–23,28,29,31,32^. The glutamine synthetase shows biosynthetic and γ-glutamyl transferase activities, which are regulated by the conversion of adenylylated and non-adenylylated forms^33–35^. The biosynthetic activity can be significantly reduced by adenylylated form while the γ-glutamyl transfer activity exists in both forms^36^. The biosynthetic activity of GS is catalyze the reaction of glutamate and ammonia to form glutamine and the γ-glutamyl transfer activity catalyze the transfer of γ -glutamyl moieties to water or amino acids, or peptides^6^. GS are usually categorized as GSII, GSIII, and GSI^37^. GSI enzymes specially exist in prokaryotes, and their structures are dodecameric^38,39^ but recently GSI enzymes have also been identified in mammals and plants^2,40^. GSI enzymes are classified into two subdivisions^37^, GSIα and GSIβ. GSIα genes are found in the thermophilic bacterium, *Thermotoga maritima* and the Euryarchaeota. GSIβ type genes exist in other bacteria such as *E*. *coli*, *Synechocystis PCC6803*, *Aquifex aeolicus*, *Crenarcheon* **s**p., and *Sulfolobus acidocaldarius*^37^.

In this study, a GSIβ glutamine synthetase protein structure from *Salmonella typhimurium* (PDB ID: 1F1H) was adapted as a homology model to build up *Pve*GS structure^41^. This dodecameric structure was in complex with two ammonium analogues, thallous ion (Tl^+^473, Tl^+^474), two manganese ion and a vital substrate ADP. Tl^+^473 coincides with the ammonium substrate binding site reported by Liaw *et al*.^4^, and the Tl^+^474 is the binding site of the ammonium group of the substrate glutamate^41^. Eisenberg has reported that hydroxylamine in the transfer reaction, ammonium ion in the biosynthetic reaction, and water in the glutamine hydrolysis reaction presumably bind at the same site^38^.

Coupling the structural information with efficient site-directed mutagenesis, the microbial expression techniques enabled successful engineering of a protein with the desired characteristics^21,23,42,43^. The objective of the present study was to obtain a mutant of glutamine synthetase obtained from a marine bacterium *P*. *vermicola*. The gene has been successfully cloned expressed and characterized to study its biochemical properties. A mutant S54A with improved catalytic efficiency, cold adaptability and higher thermostability was obtained by site-directed mutagenesis based on homology modelling of *Pve*GS. The mutant with improved catalytic features can be exploited for synthetic reactions at industrial scale.

## Results

### Gene cloning and sequence analysis

**Figure 1:**
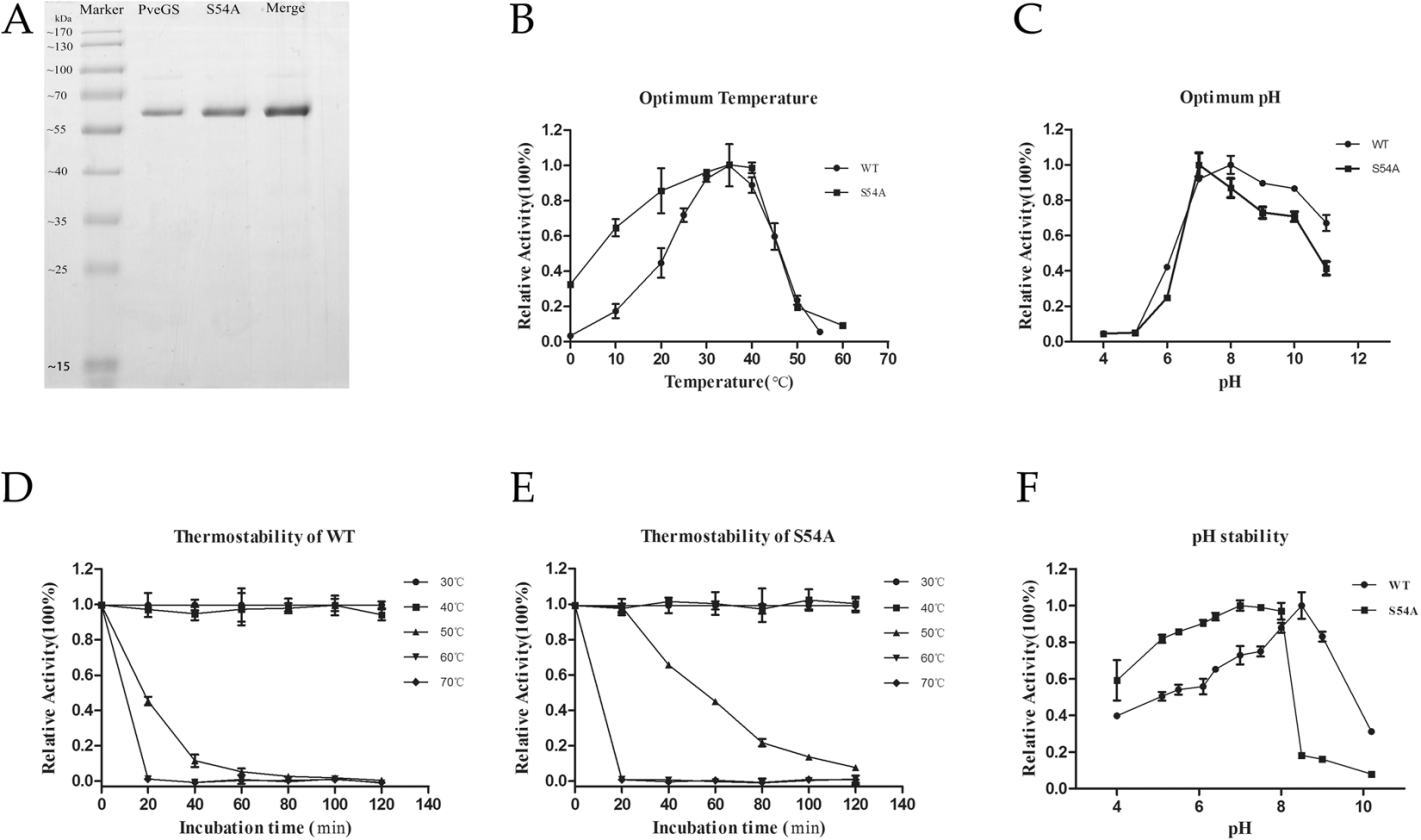
Expression and enzymatic analysis of *Pve*GS and S54A. (**A**) SDS-PAGE analysis of the purified glutamine synthetase produced in *E*. *coli* BL21. Marker lists the standard molecular weight and the lane of the wild *Pve*GS, mutant S54A and merged protein. (**B**) The optimum temperature of the wild type and S54A. The activity was determined at a gradient temperature ranging from 0 to 60 °C and activity at 35 °C was defined as 100%; (**C**) The optimum pH of the WT and S54A. The activity assay was carried out at 35 °C for 30 min and the specific activity under the optimum pH was defined as 100%. (**D**) and (**E**) Thermostability of WT and S54A was measured under different temperatures for 2 h, and samples were taken every 20 min for 2 h. The specific activity without incubation was defined as 100%. (**F**) pH stability of WT and S54A. The residual activity was measured at 35 °C for 30 min and the highest activity was defined as 100%.

The glutamine synthetase was cloned successfully from *P*. *vermicola* genomic DNA with an ORF of 1410 base pairs encoding for 469 amino acids (primers presented in Table S1). The amino acid sequence of *Pve*GS was aligned with the five- reported bacterial GS structures. Four highly conserved amino acids located in the loop region (Asp51, Tyr179, Asn24, Tyr398) and one amino acid Glu328 was located in the flap of active-site (Fig S1). Soluble *Pve*GS protein was expressed and purified from *E*. *coli* BL21 (DE3) harbouring a recombinant plasmids pGEX-6p-1-*Pve*GS. The molecular mass of the purified recombinant GS was 52 kDa after the removal of GST-tag, while its molecular mass was observed larger than expected on SDS-PAGE (Fig. 1A) due to the mino acids composition. The purified protein then loaded onto gel filtration chromatography, and the elution volume revealed that the molecular weight of *Pve*GS was more than 600 kDa (data not shown), which indicated its dodecameric structure.

### Homology modelling

Homology modelling for *Pve*GS was performed on the basis of target-template alignment, and its initial partial geometry. Heavy-atom coordinates were obtained from the conserved residues which were found between the template and *Pve*GS. The backbone coordinates were collected when residue identity was variable. Backbone geometries were modelled from fragments of high-resolution chains from the PDB (Protein Data Bank) when there were no assigned backbone coordinates according to previous library^44^. Two appropriate structural templates were from PDB among a set of pre-alignment family, with an E value of 9.8 × 10^−231^ and 7.3 × 10^−5^. The best hit template 1F1H (PDB ID) is the crystal structure of glutamine synthetase from *S*. *typhimurium*, which was adapted as modelling template after removing the first amino acid methionine of *Pve*GS. The model quality was evaluated using Ramachandran plot by MOE. The result of Ramachandran plot suggested that 462 residues were in the maximum allowable area of the plot excluding six residues (Phe81, Met98, Lys386, Lys395, Thr406 and Ser468), indicating that the model was stable in stereochemistry.

**Figure 2:**
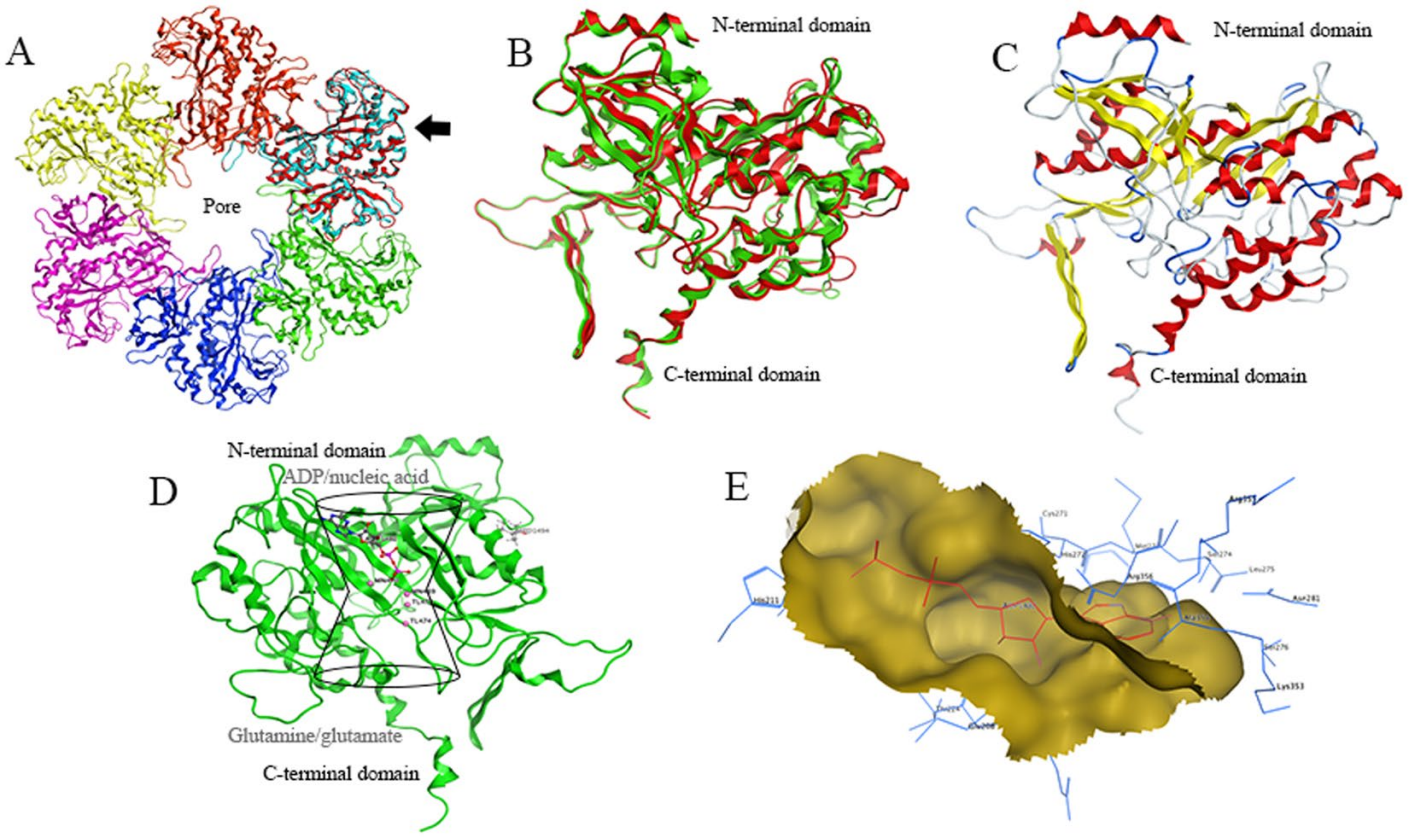
*Pve*GS model and ADP docking analysis. (**A**) Hexamer ring of *S*. *typhimurium* GS, the black arrow pointed to the superimposition of *Pve*GS and S. *typhimurium* GS monomer (cyan). (**B**) The detailed superimposition between the model of *Pve*GS and the S. *typhimurium* (green), the N terminus and C terminus domains were labelled. (**C**) The *Pve*GS homology model, with α-helices in red, and β-strands in yellow. (**D**) The interaction analysis between ADP, Tl^+^, Mn^2+^ and *Pve*GS; the schematic diagram of bifunnel structure and its substrate: nucleotide, ammonium ion, and amino acid, were noted. (**E**) The interaction surface and amino acids residues of ADP and *Pve*GS monomer.

The structure modelling and molecular weight analysis indicated that it is a dodecameric structure consists of two stacked hexamers as the typical GSI enzymes. Hydrogen bonding and hydrophobic interactions hold the two GS rings together. Each subunit possesses a C-terminus and N-terminus, in which C-terminus stabilizes the GS structure by insertion into the hydrophobic region of the subunit across the other ring. The N-terminus located on the surface and exposed to the solution environment. In addition, the central channel is formed via six four-stranded β-sheets composed of anti-parallel loops from the twelve subunits^38,45^. The *Pve*GS monomer model (red chain) superimposed to a subunit (cyan chain) of 1F1H hexamer ring were showed in Fig. 2A, as the black arrow pointing at, and different colours represented the different monomers. The superimposition between the model of *Pve*GS and the crystal structure of 1F1H from *S*. *typhimurium* (green) was presented in Fig. 2B. The homology model constructed by MOE2009 and the structure of α-helices and β-strands (yellow) was depicted in Fig. 2C.

### Ligand interaction analysis

**Figure 3:**
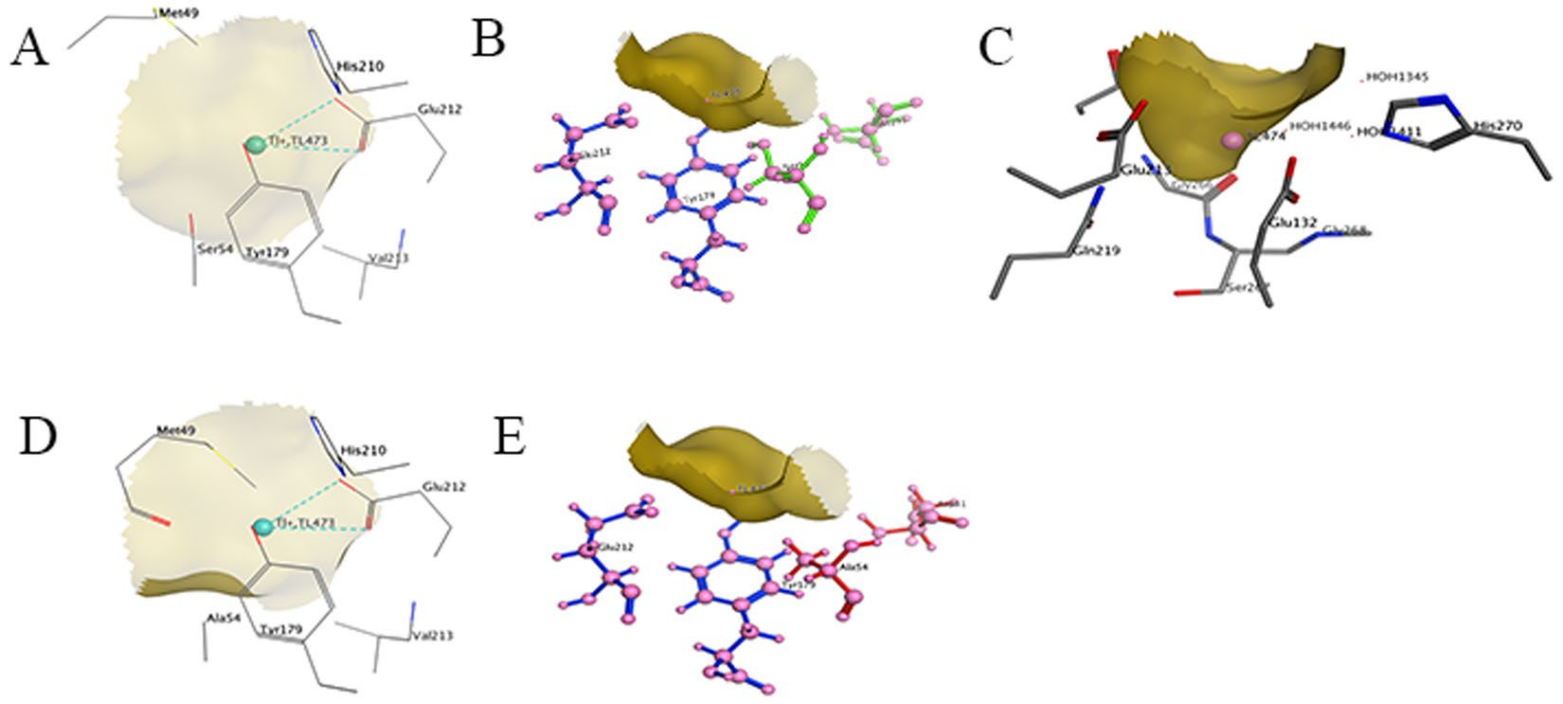
Ligand interaction analysis of the ammonium-binding site and Ser54. (**A**) and (**B**) The interaction surface of the negatively charged pocket formed by Ser 54, Asp 51, Glu 212′, and Tyr 179′ with Tl^+^473 ion. (**C**) The interaction of the negatively charged binding site of Tl^+^ 474 with the amino acid residuals, Mn^2+^ and H_2_O. (**D**) and (**E**) The Tl^+^473 interaction surface and Met49 conformational change caused by S54A.

Every *Pve*GS monomer possessed an active site named ‘bifunnel’ (Fig. 2D), which is the binding site of three distinct substrates: nucleotide, ammonium ion, and amino acid^4,46,47^. The bifunnel top was the binding site for ATP, ADP as well as nucleic acids, and glutamine, glutamate, together with ammonium bind to the bottom region. As demonstrated in Fig. 2D, ADP located in the upside of bifunnel and two ammonium analogues, thallous ion, and ammonia group of glutamate binding sites, were in the lower end. Ligand interaction revealed that ADP molecules located in a pocket formed by Asp50′ (from neighbour chain), Cys90, Ile92, Glu94, Lys170, Tyr180, Pro182, Glu221, Val222 and Glu293 (Fig. 2E). Tl^+^473 interacts with Met49, Ser54, Tyr179′, His210, Glu212 and Val213 (Fig. 3A) in which Ser54, Asp 51, Glu212 and Tyr179′ (Fig. 3B) form a negatively charged ammonium pocket. The ammonium ion could occupy this pocket and donate hydrogen bonds, similar to that of the GS model from *S*. *typhimurium* as described by Liaw *et al*.^4^. Tl^+^474 located in the negatively charged pocket for binding the amino group of the glutamate in the biosynthetic reaction, interacts with Glu132, Glu213, Gln219, Gly266, Ser267, Gly268 and His270 (Fig. 3C).

Based on the homology model, interaction analysis and sequence alignment, site-directed mutagenesis was performed on V15A, A36T, S54A, L126F, E213L, T224A, A249V and N265A. Among the generated mutants, S54A showed increased activity than wild type as well other mutants and thus selected for further investigation. The homology model was constructed for mutant GS S54A using the same template and the predicted E value was 2.2 × 10^−230^. From the homology model, it suggested that about 10 residues are out of the maximum allowable area, but it is still stable in stereochemistry. After site-directed mutagenesis in site Ser54, it was changed into Ala54 and the conformational analysis and ligand interaction of Tl^+^473 enclosed by amino acids was presented in Fig. 3D,E.

### Effects of temperature and pH on enzyme activity and stability

The relative activity of the native (*Pve*GS) and mutant (S54A) at various temperatures was presented in Fig. 1B. The optimal temperatures for the native and mutant enzymes were 35 °C and their activities were defined as 100%. *Pve*GS activity declined sharply over 40 °C and showed no activity at 0 °C, but S54A retained ~100% activity at 40 °C, 64% at 10 °C and 35% at 0 °C. This indicated that the mutant has been adopted to show activity in the cold conditions *i*.*e*. 0 and 10  °C. In the thermo-stability test, the wild and mutant enzymes retained over 95% activity after incubation at 40 °C for 2 hours. The activity of WT was decreased sharply after 40 min of incubation at 50 °C, however, S54A retained 50% activity after 60 min. (Fig. 1D,E). The native and mutant enzymes had the optimum pHs of 8.0 and 7.0 respectively (Fig. 1C). In the pH stability test at pH 11 the mutant S54A and native can retains 60 and 40% activities respectively. Additionally, S54A exhibited a respectable stability at the acidic condition (pH 4.0), retaining 60% activity as compared to 40% for the native *Pve*GS (Fig. 1F).

### Effects of metal ions and chemical reagents

These effects of metal ions and chemical reagents were determined to check their activation effects and stability of enzyme. The activity of wild type enzyme was sharply increased by Mn^2+^, NH_4_^+^ (1 and 5 mM) and Na^+^ (5 mM) and moderately increased by K^+^, Mg^2+^, Cu^2+^, Co^2+^, Li^+^, Ni^2+^ and Na^+^ at tested concentrations. The mutant S54A, has shown highest activity in the presence of Mn^2+^ (1 and 5 mM) followed by the other metal ions. But the activity of wild type was inhibited in the presence of Ba^2+^, and Fe^3+^ (Table S2). S54A was highly stable at 5 mM Ni^2+^, Li^+^, K^+^, Ba^2+^ and Mg^2+^, retaining158.9, 142.3, 141.3, 141.6 and 131.7% activity respectively, followed by Na^+^, NH_4_^+^, Co^2+^, Cu^2+^ and Zn^2+^, while the wild type enzyme showed about 100% activity only at 5 mM Ni^2+^, Li^+^, K^+^, Ba^2+^ and Mg^2+^. The activity of both the enzymes was completely inhibited in the presence of 5 mM EDTA (Table S3).

### Effects of detergents

To determine the potential industrial scale application of *Pve*GS, its tolerance towards various detergents was analysed (Table S4). These results indicated that the WT enzyme has better tolerance than S54A, indicating S54A was more sensitive to the solution environment. The wild type enzyme noted the highest relative activity of 160 and 154% in 5 mM Triton X-100 and Tween 80, respectively, higher than that in the presence of non-ionic detergents such as 5 mM Tween 20 and 0.5% CHAPS as well as 5 mM cationic surfactant CTAB. However, the anionic surfactant SDS inhibited the activity at the concentration of 0.1 and 0.5% when compared to the control. Meanwhile, the mutant activity decreased to about 18 to 40% relative to that of the control and the native. The activity of native enzyme was also inhibited in the presence of 0.1 and 0.5% SDS.

### Kinetic measurements

The enzyme kinetic parameters for the wild type and mutant enzymes were presented in Table 1. The kinetic analysis indicated that the *K*_m_ of *Pve*GS against hydroxylamine, ADP-Na_2_ and L-Glutamine were (15.7 ± 1.1), (25.2 ± 1.5) × 10^−5^ and (32.6 ± 1.7) mM, and the *k*_cat_ were (17.0 ± 0.6), (9.14 ± 0.12) and (30.5 ± 1.0) s^−1^ respectively. The kinetic analysis of mutant S54A suggested that the *K*_m_ of *Pve*GS against hydroxylamine, ADP-Na_2_ and L-Glutamine were (4.6 ± 0.3), (10.2 ± 5.6) × 10^−6^ and (23.8 ± 1.2) mM, and the *k*_cat_ were (19.6 ± 0.5), (15.2 ± 0.2) and (41.9 ± 1.1) s^−1^ respectively. The catalytic efficiency against hydroxylamine, ADP-Na_2_ and L-Glutamine of the mutant S54A was increased by 3.1-, 0.89 and 2.9-folds respectively, as compared to wild type *Pve*GS,

**Table 1:** Kinetic parameters of the wild type *Pve*GS and mutant S54A.

	ADP-Na2		L-Glutamine		Hydroxylamine	
	WT	S54A	WT	S54A	WT	S54A
*K*_m_(mM)	(25.2 ± 1.5) × 10^−5^	(10.2 ± 5.6) × 10^−5^	32.6 ± 1.7	23.8 ± 1.2	15.7 ± 1.1	4.6 ± 0.3
*V*_max_(mM/min)	0.20 ± 0.00	0.16 ± 0.00	0.66 ± 0.02	0.44 ± 0.01	0.37 ± 0.01	0.20 ± 0.01
*k*_cat_(s^−1^)	9.14 ± 0.12	15.2 ± 0.2	30.5 ± 1.0	41.9 ± 1.1	17.0 ± 0.6	19.6 ± 0.5
*k*_cat_/*K*_m_(s^−1^∙mM^−1^)	(3.63 ± 0.17) × 10^4^	(1.49 ± 0.80) × 10^5^	0.936 ± 0.02	1.76 ± 0.04	1.08 ± 0.04	4.27 ± 0.17

Kinetic parameters of the wild type and mutant S54A.

All the assays were carried out at the optimum pH and temperature for 30 min and data was given as mean values ± S.D.

## Discussion

In the present study, both the native and site-directed-mutagenesis-modified glutamine synthetases from marine *P*. *vermicola* were investigated for their catalytic properties under optimized conditions. The native (*Pve*GS) and mutant (S54A) enzymes were characterized in terms of optimum temperature, pH, and thermal stability. The *Pve*GS and S54A were found to be highly active at 40 °C for 2 h, which is similar to the previously reported GSI of *P*. *ruminicola*^48^. The mutant S54A showed a relatively high residual activity, about 64 and 35% at 10 °C and 0 °C respectively. These findings have been supported by a previous study similar to the wild esterase and lipase reported from *Psychrobacter* sp. Ant 300 and *Pseudomonas* sp.732^49,50^. As reported previously, the low ratio of Arginine/(Arginine + Lysine), low proportion of proline residues, small hydrophobic core, lesser salt bridges and aromatic–aromatic interactions are the common characteristics for a cold-adapted enzyme^51–54^. A smaller number of ion pairs and weak charge-dipole interactions in α- helices could contribute to the poor thermo-stability^55^. In the present investigation, S54A was located on the loop of the active site, which leads to more plasticity and flexibility to the structure. Meanwhile, the hydrogen bonds between the product and enzyme was destroyed, which is facilitating the release of the substrate at a lower temperature. The cold-adapted enzyme is a promising source to the pharmaceutical industries, agriculture and chemical industries^56^, for cold conditions are required for biosynthesis of fragile pharmaceutical compounds to avoid adverse side-effects and conserve energy^54^.

Glutamine synthetase is a metallo-protein possessing a metal binding site in its active site to accommodate the cofactors (Mg^2+^ or Mn^2+^) that are important for its activity. Cations binds to the metal binding site of the enzyme as previously reported^57^. A previous study revealed that there were mono-valent cation sites in GS so that these cations could stabilize the quaternary structure of GS and alkali ions could compete partially with NH_2_OH in the γ-glutamyl transfer reaction^4^. *Pve*GS and S54A exhibited a relatively low residual activity (6.1 and 4.4%) in the presence of 5 mM EDTA, because the combination of Mn^2+^ with EDTA resulted in the lack of activating metal ions^58^. The presence of cationic detergent (CTAB) and anion detergent (SDS) can break the non-covalent bond between the proteins, leading to the decrease of the activity. Non-ionic interaction between the protein and lipid separated, and the relative residual activity of *Pve*GS and S54A was different at a higher concentration, which was like a previous report about the glutamine synthetase from *Azospirillum brasilense*^58^.

The ligands to the Tl^+^ 473 ion are shown as atomic models in which Ser 54, Asp 51, Glu 212′, and Tyr 179′ (from the neighbouring chain) form a negatively charged ammonium pocket, and the ammonium ion could occupy the site and donate hydrogen bonds, which is similar to a previously described model of GS from *S*. *typhimuriu*^4^. Then, the negatively charged pocket forms the binding site of Tl^+^ 474 to bind the amino group of the glutamate. Both the negatively charged pockets were situated on the same half side of the bottom of the bifunnel. The other half side of the bottom, which is positively charged, stabilizes the R- and γ-carboxylate groups of the glutamate in biosynthetic activity as well as the glutamine in γ-glutamyl transfer activity^41^.

After site-directed mutagenesis in site Ser54, was mutated into Ala54, and the conformational analysis indicated that the mutation destroys the side chain interaction between Gly56 and Ser54 as well as the receptor contact of Tl^+^473, which was the ammonium group of the substrate-binding site of hydroxylamine and cofactor ADP^4^. Meanwhile, the exposure of the ligand atoms was also changed. The substrate hydroxylamine bound with the negatively charged ammonium pocket where the binding of S54A was not as strong as that of the wild type. The ammonium group could transfer to the glutamine with less effective hydrogen bonds, resulting in higher catalytic efficiency. On the other hand, the S54A has changed the hydrogen bonds to the L-Glutamic acid γ-monohydroxamate, thus improving the catalytic efficiency for different types of substrates.

## Material and Methods

### Bacterial strains and plasmid

The marine bacterium *P*. *vermicola* strain CGS6 was obtained from Marine Culture Collection of China (http://www.mccc.org.cn/). *P*. *vermicola* was grown in nutrient agar medium containing peptone (1%, w/v), yeast extract (0.5%, w/v), NaCl (2%, w/v), and agar (1.5%, w/v). Plasmid pGEX-6p-1 (GE Healthcare, USA) was used as a vector for protein expression. For cloning and expression, *E*. *coli* DH5α (TaKaRa, Japan) and *E*. *coli* BL21 (DE3) (Novagen, USA) were used respectively. These bacteria were cultured in Luria-Bertani medium.

### Structural modelling

To obtain a suitable template for *Pve*GS homology modelling, search command performed on a database of protein structures and sequences that had been clustered into families was performed by Swiss-Model (http://swissmodel.expasy.org) as well as PDB search in MOE 2009. Based on the pre-align family sequences, the alignment done to decide the template. Stereochemistry of homology models was calculated to evaluate if there were unusual or geometrically unreasonable features present. The superimposition of GS with the template and both the catalytic residues were generated by the MOE through the “superpose”.

### Cloning and Site-directed mutagenesis

The *Pve*GS gene was amplified using *Pve*GS-F and *Pve*GS-R primers (Table S1) containing *Bam*HI and *Xho*I restriction sites respectively. The genomic DNA of *P*. *vermicola* was used as template. Then the target gene was cloned into pGEX-6P-1 vector by *Bam*HI and *Xho*I dual digestion and T4 DNA ligase ligation. The ligation product was transferred into *E*. *coli* DH5α competent cells for screening of positive recombinant. The recombinant plasmid pGEX-6P-1-*Pve*GS was used as a template for the PCR with the primers (Table S1) to perform site-directed mutagenesis. The PCR products were digested with *Dpn*I to remove the template plasmids, and subsequent experiments were carried out as mentioned above.

### Expression and purification

*E*. *coli* BL21 (DE3) cells harbouring pGEX-6p-1-*Pve*GS were inoculated into LB broth with 100 μg/ml ampicillin. The culture was induced by adding 0.15 mM IPTG into when the OD600 was 0.6–0.8, then incubated at 18 °C for 16 h under shaking (225 rpm/min). Finally, the induced cells were collected by centrifugation, resuspended in phosphate-buffered saline (PBS) buffer (NaCl 0.8%, KCl 0.02%, Na_2_HPO_4_ 0.14%, KH_2_PO_4_ 0.03%; pH 7.0) and homogenized using a high-pressure homogenizer (NS100IL 2 K, Niro Soavi, Germany). PveGS recombined with GST-tag were purified using a glutathione S-transferase (GST) Gene Fusion System (GE Healthcare, USA) and eluted from the GST tag by 3 C proteases (PreScission, Pharmacia). The protein was quantified using the Bradford reagent with bovine serum albumin (BSA) as a standard^59^ and the molecular weight was confirmed by sodium dodecyl sulfate-denatured polyacrylamide gel electrophoresis (SDS-PAGE) with 12% polyacrylamide gels. Then the tag-free protein sample was concentrated to load the gel filtration chromatography column “Superose 6, 10/300 GL” with regular buffer (25 mM Tris-HCl pH 7.5, 150 mM NaCl).

### Assay of glutamine synthetase activity

The GS activity assay contained 18 mM hydroxylamine-hydrochloride, 20 mM L-Glutamine, 1 mM MnCl_2_, 25 mM potassium arsenate, 0.4 mM ADP-Na_2_, and 135 mM imidazole hydrochloride. The reaction was terminated by adding 200 µL ‘stop mixture’ consisting of 55 g FeCl_3_▪6H_2_O, 20 g trichloroacetic acid and concentrated HCl (21 mL/L). The absorbance was measured at 540 nm after centrifugation of reaction mixture^36,60^. The L-Glutamic acid γ-monohydroxamate was added into the reaction mixture without enzyme to give a concentration from 0 to 6 mM for quantification by the absorbance at 540 nm. One unit of enzyme activity is defined as the amount of *Pve*GS required convert 1µmol L-Glutamic acid γ-monohydroxamate in one minute in one ml of reaction mixture under optimized conditions.

### Effect of temperature and pH

The assay mixture and enzyme were incubated in the temperature gradient from 0 to 60 °C for 5 min and the reaction was initiated by adding L-Glutamine and terminated after 30 min incubation using stop reagent described in previous sections. The thermostability assay was conducted in the temperature gradient from 30 to 70 °C and the samples were taken after every 20 min for 2 h. The residual activity was determined under the optimized conditions. Phosphate-citrate (pH 4.0–8.0) and Glycine-NaOH (pH 8.0–11.0) buffer were used to analyze the effect pH of 4.0 to 8.0 and 8.0 to 11.0, respectively. pH stability was determined by calculating the residual activity left after incubating the enzyme for 24 hours at 4 °C.

### Effect of metal ions and detergents on the native and mutant GS activity

The effects of various metal ions (K^+^, Mg^2+^, Mn^2+^, Zn^2+^, Cu^2+^, Ba^2+^, Ni^2+^, Fe^3+^, Co^2+^, Li^+^, NH_4_^+^, Na^+^), reagents (EDTA, Urea, DTT, PMSF) and detergents (Tween 20, Tween 80, Triton X-100, CHAPS, CTAB, SDS) on the enzymatic activity were determined by using the different concentrations of reagents (1 and 5 mM) and detergents (1 and 5 mM, and 0.1 and 0.5%, v/v). The effects of metal ions K^+^, Mg^2+^, Mn^2+^, Zn^2+^, Cu^2+^, Ba^2+^, Ni^2+^, Fe^3+^, Co^2+^, Li^+^, NH_4_^+^ and Na^+^ on the activity were tested by adding them into the reaction mixture without MnCl_2_ at the concentration of 1 and 5 mM. The enzyme stability was tested with 2 mM MnCl_2_ and 1 or 5 mM of various reagents. The relative activity was determined by comparing to the control.

### The kinetic parameters *K*_*m*_ and *k*_*cat*_

The substrates of the *Pve*GS, L-Glutamine, hydroxylamine-hydrochloride and ADP-Na_2_ were added into the mixture with the concentration of 2–40 mM, 0.05–20 mM and 0.00001–0.01 mM, respectively, to obtain the initial rate of reaction. Michaelis-Menten constant^61^ was measured according to Lineweaver–Burkplot, and the value of *V*_max_, the molecular mass and the concentration of the purified protein were used to calculate the catalytic constant (*k*_*cat*_). Graphpad Prism software (Graphpad, San Diego, CA, USA) was also used to calculate the parameters for consistency and accuracy.

### Molecular docking

To search for the active site and investigate the interaction surface of the GS, molecular docking was performed. After obtaining the homologous sequences with a reasonable value, the structure containing the ligand was selected as the target substrate for docking. In present study, a GSIβ glutamine synthetase protein structure from *S*. *typhimurium*^41^ (PDB ID: 1F1H) was adapted, which containing ligand like ADP, and two important ammonium analogues, Tl^+^, which were coincidence with ammonium substrate binding site and binding the ammonium group of the glutamate. Based on the homology modelling to WT and S54A, molecular docking analysis was carried out on the instruction of docking tutorial in MOE 2009.

## Electronic supplementary material

supplementary information

## References

[1] Somvanshi VS (2006). Providencia vermicola sp nov., isolated from infective juveniles of the entomopathogenic nematode Steinernema thermophilum. Int J Syst Evol Micr.

[2] Wyatt K (2006). Lengsin is a survivor of an ancient family of class I glutamine synthetases re-engineered by evolution for a role in the vertebrate lens. Structure.

[3] Eisenberg D (1987). Some evolutionary relationships of the primary biological catalysts glutamine synthetase and RuBisCO. Cold Spring Harbor symposia on quantitative biology.

[4] Liaw SH, Kuo I, Eisenberg D (1995). Discovery of the ammonium substrate site on glutamine synthetase, a third cation binding site. Protein science: a publication of the Protein Society.

[5] Hirel B, McNally SF, Gadal P, Sumar N, Stewart GR (1984). Cytosolic glutamine synthetase in higher plants. A comparative immunological study. European journal of biochemistry/FEBS.

[6] Al-Gharawi A, Moore D (1977). Factors affecting the amount and the activity of the glutamate dehydrogenases of Coprinus cinereus. Biochimica et biophysica acta.

[7] Ebner E, Wolf D, Gancedo C, Elsasser S, Holzer H (1970). ATP: glutamine synthetase adenylyltransferase from Escherichia coli B. Purification and properties. European journal of biochemistry/FEBS.

[8] El Alaoui S (2003). Glutamine synthetase from the marine cyanobacteria Prochlorococcus spp: characterization, phylogeny and response to nutrient limitation. Environmental microbiology.

[9] Yin ZM, Chen QY, Sima J, Wu YF, Zhang SQ (2003). The expression regulation and characterization of glutamine synthetase from the hyperthermoacidophilic crenarcheon Sulfolobus acidocaldarius. Prog Biochem Biophys.

[10] Liaw SH, Eisenberg D (1994). Structural model for the reaction mechanism of glutamine synthetase, based on five crystal structures of enzyme-substrate complexes. Biochemistry.

[11] Abell LM, Villafranca JJ (1991). Investigation of the mechanism of phosphinothricin inactivation of Escherichia coli glutamine synthetase using rapid quench kinetic technique. Biochemistry.

[12] Zhang S (2017). Characterization of an L-phosphinothricin resistant glutamine synthetase from Exiguobacterium sp. and its improvement. Applied microbiology and biotechnology.

[13] Wakisaka S, Ohshima Y, Ogawa M, Tochikura T, Tachiki T (1998). Characteristics and efficiency of glutamine production by coupling of a bacterial glutamine synthetase reaction with the alcoholic fermentation system of baker’s yeast. Applied and environmental microbiology.

[14] Yamamoto S, Wakayama M, Tachiki T (2005). Theanine production by coupled fermentation with energy transfer employing Pseudomonas taetrolens Y-30 glutamine synthetase and baker’s yeast cells. Bioscience, biotechnology, and biochemistry.

[15] Yamamoto S, Wakayama M, Tachiki T (2006). Cloning and expression of Pseudomonas taetrolens Y-30 gene encoding glutamine synthetase: an enzyme available for theanine production by coupled fermentation with energy transfer. Bioscience, biotechnology, and biochemistry.

[16] Yokogoshi H, Kobayashi M, Mochizuki M, Terashima T (1998). Effect of theanine, r-glutamylethylamide, on brain monoamines and striatal dopamine release in conscious rats. Neurochemical research.

[17] Sugiyama T, Sadzuka Y (2003). Theanine and glutamate transporter inhibitors enhance the antitumor efficacy of chemotherapeutic agents. Biochimica et biophysica acta.

[18] Zhou X (2008). Mn(2+) enhances theanine-forming activity of recombinant glutamine synthetase from Bacillus subtilis in Escherichia coli. World J Microb Biot.

[19] Yokoyama T (2011). Synthesis of l-theanine using enzyme/mesoporous silica conjugates under high pH conditions. Materials letters.

[20] Itoh T (2012). Production of l-theanine using glutaminase encapsulated in carbon-coated mesoporous silica with high pH stability. Biochemical engineering journal.

[21] Zhang S, Wu G, Feng S, Liu Z (2014). Improved thermostability of esterase from Aspergillus fumigatus by site-directed mutagenesis. Enzyme and microbial technology.

[22] Packer MS, Liu DR (2015). Methods for the directed evolution of proteins. Nature Reviews Genetics.

[23] Yao P (2015). Improvement of glycine oxidase by DNA shuffling, and site-saturation mutagenesis of F247 residue. International journal of biological macromolecules.

[24] Chen J, An Y, Kumar A, Liu Z (2017). Improvement of chitinase Pachi with nematicidal activities by random mutagenesis. International journal of biological macromolecules.

[25] Dome JS (1999). High telomerase reverse transcriptase (hTERT) messenger RNA level correlates with tumor recurrence in patients with favorable histology Wilms’ tumor. Cancer research.

[26] Anwar MZ (2017). SnO2 hollow nanotubes: a novel and efficient support matrix for enzyme immobilization. Scientific reports.

[27] Kumar A (2015). Cellulose binding domain assisted immobilization of lipase (GSlip–CBD) onto cellulosic nanogel: characterization and application in organic medium. Colloids and Surfaces B: Biointerfaces.

[28] Chen L, Chen J, Kumar A, Liu Z (2015). Effects of domains modification on the catalytic potential of chitinase from Pseudomonas aeruginosa. International journal of biological macromolecules.

[29] Kumar Ashok, Wu Gaobing, Wu Zuo, Kumar Narendra, Liu Ziduo (2018). Improved catalytic properties of a serine hydroxymethyl transferase from Idiomarina loihiensis by site directed mutagenesis. International Journal of Biological Macromolecules.

[30] Wu G, Zhan T, Guo Y, Kumar A, Liu Z (2016). Asn336 is involved in the substrate affinity of glycine oxidase from Bacillus cereus. Electronic Journal of Biotechnology.

[31] Kim, J., Kim, S., Yoon, S., Hong, E. & Ryu, Y. Improved enantioselectivity of thermostable esterase from Archaeoglobus fulgidus toward (S)-ketoprofen ethyl ester by directed evolution and characterization of mutant esterases. *Applied microbiology and biotechnology*, 1–9 (2015). 10.1007/s00253-015-6422-7 25661815

[32] Chronopoulou EG, Labrou NE (2011). Site‐saturation Mutagenesis: A Powerful Tool for Structure‐Based Design of Combinatorial Mutation Libraries. Current Protocols in Protein Science,.

[33] Hennig SB, Anderson WB, Ginsburg A (1970). Adenosine triphosphate: glutamine synthetase adenylyltransferase of Escherichia coli: two active molecular forms. Proceedings of the National Academy of Sciences of the United States of America.

[34] Abell LM, Villafranca JJ (1991). Effect of metal ions and adenylylation state on the internal thermodynamics of phosphoryl transfer in the Escherichia coli glutamine synthetase reaction. Biochemistry.

[35] Jiang P, Mayo AE, Ninfa AJ (2007). Escherichia coli glutamine synthetase adenylyltransferase (ATase, EC 2.7.7.49): Kinetic characterization of regulation by PII, PII-UMP, glutamine, and alpha-ketoglutarate. Biochemistry.

[36] Bender RA (1977). Biochemical parameters of glutamine synthetase from Klebsiella aerogenes. Journal of bacteriology.

[37] Brown JR, Masuchi Y, Robb FT, Doolittle WF (1994). Evolutionary relationships of bacterial and archaeal glutamine synthetase genes. Journal of molecular evolution.

[38] Eisenberg D, Gill HS, Pfluegl GM, Rotstein SH (2000). Structure-function relationships of glutamine synthetases. Biochimica et biophysica acta.

[39] Almassy RJ, Janson CA, Hamlin R, Xuong NH, Eisenberg D (1986). Novel subunit-subunit interactions in the structure of glutamine synthetase. Nature.

[40] Mathis R, Gamas P, Meyer Y, Cullimore JV (2000). The presence of GSI-like genes in higher plants: Support for the paralogous evolution of GSI and GSII genes. Journal of molecular evolution.

[41] Gill HS, Eisenberg D (2001). The crystal structure of phosphinothricin in the active site of glutamine synthetase illuminates the mechanism of enzymatic inhibition. Biochemistry.

[42] Blundell TL, Sibanda BL, Sternberg MJ, Thornton JM (1987). Knowledge-based prediction of protein structures and the design of novel molecules. Nature.

[43] Sánchez Roberto, Šali Andrej (1997). Evaluation of comparative protein structure modeling by MODELLER‐3. Proteins: Structure, Function, and Genetics.

[44] Berman HM (2000). The Protein Data Bank. Nucleic Acids Res.

[45] Yamashita MM, Almassy RJ, Janson CA, Cascio D, Eisenberg D (1989). Refined atomic model of glutamine synthetase at 3.5 A resolution. The Journal of biological chemistry.

[46] Ginsburg A, Yeh J, Hennig SB, Denton MD (1970). Some effects of adenylylation on the biosynthetic properties of the glutamine synthetase from Escherichia coli. Biochemistry.

[47] Krajewski WW (2008). Crystal structures of mammalian glutamine synthetases illustrate substrate-induced conformational changes and provide opportunities for drug and herbicide design. Journal of molecular biology.

[48] Kim JN, Cann IKO, Mackie RI (2009). Purification, Characterization, and Regulation of Glutamine Synthetase from Prevotella ruminicola 23. Microb Ecol.

[49] Kulakova L, Galkin A, Nakayama T, Nishino T, Esaki N (2004). Cold-active esterase from Psychrobacter sp. Ant300: gene cloning, characterization, and the effects of Gly–>Pro substitution near the active site on its catalytic activity and stability. Biochimica et biophysica acta.

[50] Zhang JW, Zeng RY (2008). Molecular cloning and expression of a cold-adapted lipase gene from an Antarctic deep sea psychrotrophic bacterium Pseudomonas sp. 7323. Marine biotechnology.

[51] Wu G (2015). A cold-adapted, solvent and salt tolerant esterase from marine bacterium Psychrobacter pacificensis. International journal of biological macromolecules.

[52] Rahman MA (2016). Characterization of a novel cold active and salt tolerant esterase from Zunongwangia profunda. Enzyme and microbial technology.

[53] Rahman MA, Culsum U, Kumar A, Gao H, Hu N (2016). Immobilization of a novel cold active esterase onto Fe 3 O 4∼ cellulose nano-composite enhances catalytic properties. International journal of biological macromolecules.

[54] Joseph B, Ramteke PW, Thomas G (2008). Cold active microbial lipases: some hot issues and recent developments. Biotechnology advances.

[55] Feller G, Gerday C (2003). Psychrophilic enzymes: hot topics in cold adaptation. Nature reviews. Microbiology.

[56] Panda T, Gowrishankar BS (2005). Production and applications of esterases. Applied microbiology and biotechnology.

[57] Gomez-Baena G, Dominguez-Martin MA, Donaldson RP, Garcia-Fernandez JM, Diez J (2015). Glutamine Synthetase Sensitivity to Oxidative Modification during Nutrient Starvation in Prochlorococcus marinus PCC 9511. PloS one.

[58] Kamnev AA (2004). Structural characterization of glutamine synthetase from Azospirillum brasilense. Biopolymers.

[59] Bradford MM (1976). A rapid and sensitive method for the quantitation of microgram quantities of protein utilizing the principle of protein-dye binding. Analytical biochemistry.

[60] Shapiro BM, Stadtman ER (1970). The regulation of glutamine synthesis in microorganisms. Annual review of microbiology.

[61] Rockmill B, Roeder GS (1998). Telomere-mediated chromosome pairing during meiosis in budding yeast. Genes & development.

