# Supplementary material for: Characterization and improved properties of Glutamine synthetase from Providencia vermicola by site-directed mutagenesis
Source: Sci Rep. 2018 Oct 23;8:15640. doi: 10.1038/s41598-018-34022-5 (PMC6199252; doi:10.1038/s41598-018-34022-5)
Supplement: Supplementary file 1 — supplementary information [file 41598_2018_34022_MOESM1_ESM.doc]

**Supplementary material**

# Characterization and improved properties of Glutamine synthetase from *Providencia vermicola* by site-directed mutagenesis

Wu Zuo^2, †^, Leitong Nie^2, †^, Ram Baskaran^2^, Ashok Kumar^3^, Ziduo Liu^1, 2*^

*^1^* *College of Food and Bio-science and Technology,* *Wuhan Institute of Design and Science, Wuhan, P.R. China, 430205*

*^2^College of Life Science and Technology, State Key Laboratory of Agricultural Microbiology, Huazhong Agricultural University, Wuhan, P.R. China, 430070*

*^3^Department of Biotechnology and Bioinformatics, Jaypee University of Information Technology,* *Waknaghat, Solan* *173234, Himachal Pradesh, India*

^†^These authors contributed equally to this work.

***Correspondence:** **Ziduo Liu** lzd@mail.hzau.edu.cn

**Supplementary Table S1** Primers for species determination, the gene encoding GS and site-directed mutagenesis.

**Supplementary Table S2.** Effect of various activating agents on GS activity

**Supplementary Table S3.** Effect of various metal ions on GS activity

**Supplementary Table S4.** Effect of detergents on GS activity

**Supplementary Fig S1**. Multiple sequence alignment of *Pve*GS and other glutamine synthetase genes. Residues in the red boxes are corresponding active sites. *Salmonella typhimurium* (GI:9256961), *Escherichia coli* K12 (GI:764144519), *Bacillus subtilis* (GI:764091079), *Corynebacterium glutamicum* (GI:499324468), *Mycobacterium tuberculosis* (GI:22218713). The red highlighted region indicated four active site loops: Asp51 loop, Tyr179 loop, Asn245 loop, Tyr398 loop and Glu328 flap.

**Supplementary Table S1 Primers for species determination, the gene encoding GS and site-directed mutagenesis.**

| Primer | Sequence (5’–3’) |
| --- | --- |
| *16s rDNA-F* | TGACGAGTGGCGGACGGGTG |
| *16s rDNA-R* | CACACCGCCCGTCACACCATGG |
| *Pve*GS-F | CGCGGATCCATGTCCGCTGAACATGTTTTATCGTTAATTA |
| *Pve*GS-R | CCGCTCGAGTTACACACTGTAGTACATTTCAAATTCGAGA |
| S54A-F | TGAAGAAGGCCAGATGTTTGATGGTTCA**G** |
| S54A-R | CCACCAATAG**C**TGAACCATCAAACATCTG |

The restriction endonuclease sites *Bam*HI/*Xho*I are underlined and the altered base sites are bold.

**Supplementary Table S2.** Effect of various activating agents on GS activity

| Activating agents | Relative activity of WT (%) | |  |  | Relative activity of S54A (%) | |
| --- | --- | --- | --- | --- | --- | --- |
|  | Concentration  (1 mM) | Concentration  (5 mM) |  |  | Concentration  (1 mM) | Concentration  (5 mM) |
| Control  K^+^  Mg^2+^  Mn^2+^  Zn^2+^  Cu^2+^  Ba^2+^  Ni^2+^  Fe^3+^  Co^2+^  Li^+^  NH_4_^+^  Na^+^ | 100±0.7  10.8±0.5  18.2±1.7  100±4.4  5±2.5  21.7±0.6  3.5±5.7  10.9±2.2  -0.6±1.1  28±6  36.7±0.7  49.9±5.4  31.8±4.8 | 100±6.8  28.5±4.9  13.3±2.9  70.9±8.7  4.5±2.5  32.5±0.8  -2.4±2.2  7.8±2.1  -2.7±1.8  33.9±1.6  35.5±0.2  50.8±4.7  42.1±4 |  |  | 100±3.4  4.6±0.3  9.1±0.3  100±1.4  4.3±0.1  6.3±0  4.4±0.1  4.1±0  6±0.1  6±0.1  4.4±0.2  4.6±0.2  4.2±0.1 | 100±1.7  4.1±0  12.7±0.3  63.5±6.9  4.1±0.1  7.5±0.1  3.9±0  3.9±0  5.3±0.1  7.4±0.1  4.3±0.1  4.2±0.2  4.2±0.1 |

Effects of various activating regents on the activity of *Pve*GS and S54A. Activity was determined by adding 1 and 5 mM reagent into the reaction mixture. The specific activity without any reagent was defined as 100 % and data was given as mean values ± S.D.

**Supplementary Table S3.** Effect of various metal ions on GS activity

| Reagent | Relative activity of WT (%) | |  |  | Relative activity of S54A (%) | |
| --- | --- | --- | --- | --- | --- | --- |
|  | Concentration  (1 mM) | Concentration  (5 mM) |  |  | Concentration  (1 mM) | Concentration  (5 mM) |
| Control  K^+^  Mg^2+^  Mn^2+^  Zn^2+^  Cu^2+^  Ba^2+^  Ni^2+^  Fe^3+^  Li^+^  Na^+^  NH_4_^+^  Co^2+^  EDTA  Urea  DTT  PMSF | 100±0.7  88.6±1  93.4±1.7  88.2±3.1  89.4±2.5  46.6±1.7  91.6±0.9  94.2±1.5  77±2.6  93.8±0.9  91.2±1.3  93.1±0.9  88.9±3.9  106.6±1.2  104.5±0.5  102.4±2.9  96.9±4.1 | 100±6.8  106±0.9  110±1.9  100.7±4.7  22.9±0.6  25.1±0.4  107.5±0.7  107.5±1  54.9±2.1  108.6±1.3  107.6±2.6  110±1.5  102.3±2.3  6.1±0  102.6±0.9  101±0.8  91±0.6 |  |  | 100±3.4  104.3±0.7  106.2±6.1  107.7±3.1  78.6±1.7  85.7±2.2  105.6±0.6  101.5±1.4  99.4±1.7  104.4±2.2  107.2±1.1  99±0.1  101±1.7  115.3±3.4  92.9±3.7  100.9±3.1  114.4±4.4 | 100±1.7  141.3±2.3  131.7±1.7  104.3±10.1  57±1.2  69.2±1.2  141.6±4.3  158.9±2.1  91.8±5.7  142.3±0.9  120.9±25.3  117.7±0.1  128.8±2.5  4.4±0  98.5±0.7  101.9±1.4  88±6.4 |

Effect of various reagents on GS activity. 1 mM or 5 mM reagent was added into the reaction mixture to measure the activity in the presence of 2 mM MnCl_2_. The specific activity with 2mM MnCl2 was defined as 100 % and data was given as mean values ± S.D.

**Supplementary Table S4.** Effect of detergents on GS activity

| Detergent |  | Relative activity of WT (%) | |  | Relative activity of S54A (%) | |
| --- | --- | --- | --- | --- | --- | --- |
|  |  | Concentration  (1 mM) | Concentration  (5 mM) |  | Concentration  (1 mM) | Concentration  (5 mM) |
| Control |  | 100±1 | 100±1 |  | 100±3.8 | 100±3.8 |
| Tween 20 |  | 97.8±0.9 | 118±7.6 |  | 63.2±2.7 | 66.8±1.9 |
| Tween 80 |  | 97.7±0.3 | 154.1±7 |  | 67.1±7.1 | 76±6.1 |
| TritonX-100 |  | 91.8±2 | 160.1±2.9 |  | 66.4±1.7 | 82.7±3.6 |
| CHAPS(w/v) |  | 89±2 | 102.3±3.9 |  | 72.1±2.2 | 77±6.7 |
| CTAB (w/v) |  | 102.3±9.1 | 130±4.9 |  | 84.3±3.4 | 60.1±0.1 |
| SDS (w/v) |  | 40.3±1.2 | 41.7±3.3 |  | 10.7±0.7 | 8.8±0.1 |

Effect of detergents on GS activity. The activity was determined in the presence of 1mM and 5mM (0.1 and 0.5 %) detergents. The specific activity without detergent was defined as 100 % and data was given as mean values ± S.D.


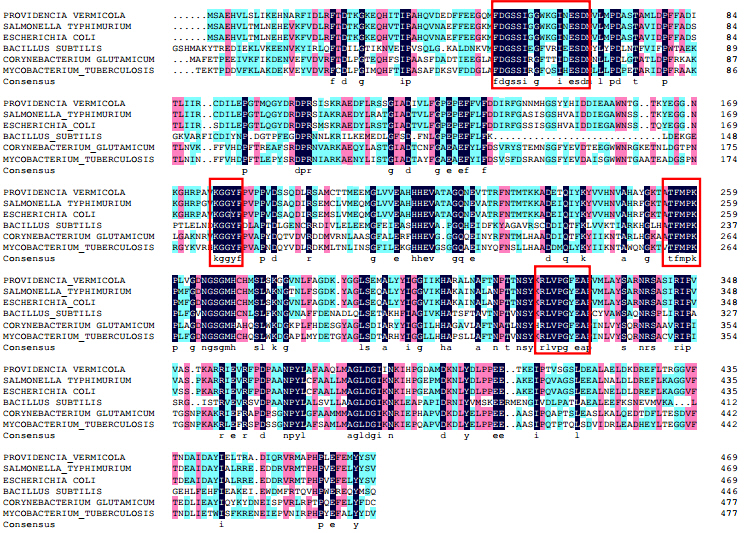


**Supplementary Fig S1**. Multiple sequence alignment of *Pve*GS and other glutamine synthetase genes. Residues in the red boxes are corresponding active sites. *Salmonella typhimurium* (GI:9256961), *Escherichia coli* K12 (GI:764144519), *Bacillus subtilis* (GI:764091079), *Corynebacterium glutamicum* (GI:499324468), *Mycobacterium tuberculosis* (GI:22218713). The red highlighted region indicated four active site loops: Asp51 loop, Tyr179 loop, Asn245 loop, Tyr398 loop and Glu328 flap.
